# Assessing Decision-Making Tools for Meaningful Discussions About Transfer Decisions From Long-Term Care to Hospital: A Scoping Review

**DOI:** 10.1177/07334648251394471

**Published:** 2025-11-14

**Authors:** Jia Qi Adam Bai, Alixe Ménard, Yamini Singh, Daniel Kobewka, Krystal Kehoe MacLeod

**Affiliations:** 1 Department of Medicine, 10055 Ottawa Hospital Research Institute, Ottawa, ON, Canada; 2 152971 Bruyère Health Research Institute, Ottawa, ON, Canada; 3 Faculty of Medicine, 12365 University of Ottawa, Ottawa, ON, Canada

**Keywords:** long-term care, patient transfer, hospitalization, decision support techniques

## Abstract

The decision-making process for transferring long-term care (LTC) residents to acute care hospitals is complex. These transfers can carry significant health risks, such as loss of function, morbidity, and mortality. Decision-making tools have emerged to guide these transfers, offering structured frameworks for informed discussions. Despite their benefits, their application remains inconsistent across LTC settings. MEDLINE, Embase, and CENTRAL were searched, following PRISMA-Scr guidelines. Studies evaluating decision support tools for LTC-to-hospital transfers were included. Of 1,383 studies identified, 15 studies involving 50,175 patients were included. Tools were categorized into five intervention types: educational booklets, decision aid videos, multidisciplinary programs, advance directive programs, and checklists. Most studies reported reduced hospitalization rates and improved communication, but variability in tool types highlighted their fragmented application. This scoping review summarizes reported outcomes and highlights gaps in the application and evaluation of decision-making tools for LTC-to-hospital transfers, emphasizing the need for more standardized and culturally sensitive interventions.

**What This Paper Adds**

• We identified and categorized five domains of decision-making tools for LTC-to-hospital transfers. We also highlighted the most effective strategies while revealing inconsistencies in outcomes.
• Uncovered the lack of resident partner involvement in tool development and the absence of culturally sensitive approaches, urging more inclusive, person-centered designs.
• Connects tool efficacy to implementation, offering a roadmap for optimizing real-world adoption of these decision-making tools.

**Applications of Study Findings**

• Supports LTC staff in adopting structured tools (e.g., checklists and advance care planning videos) to reduce avoidable transfers and improve communication during LTC-to-hospital transfer decisions.
• Advocates for standardized, adaptable toolkits in LTC regulations, with incentives for facilities to integrate resident and family voices, as well as cultural considerations.
• Underscores the need for longitudinal studies on tool sustainability and trials co-designed with residents to ensure equity and usability across diverse populations.

## Background

Although long-term care (LTC) residents receive around-the-clock care, they are commonly transferred to emergency departments (ED) to receive care for injuries, illness or exacerbation of chronic disease (Turcotte et al., 2023). Decision-making surrounding these transfers is critical and complex, requiring detailed discussions among residents, care partners and healthcare professionals to occur during times of high emotion and stress (Arendts et al., 2013). For LTC residents, many of whom are frail or cognitively impaired, transfers to hospital can significantly compromise their emotional well-being, quality of life, and health outcomes (Trahan et al., 2016). Several recent studies have reported an increase in morbidity and mortality at the individual level due to emotional distress and loss of function following ED transfers (Derlet & Richards, 2000; Hodgins et al., 2010; Savioli et al., 2022). At a system level, these hospital transfers exacerbate ED overcrowding and impose financial strain on the health care system (Grant et al., 2020; Trahan et al., 2016). Therefore, improving the transfer decision-making process to reduce unnecessary acute care transfers addresses not only financial and operational challenges but also ensures resident-centered care and patient safety (Trahan et al., 2016).

How to deal with changes in a LTC resident’s health status is often context-dependent, demanding the consideration of both the resident’s overall condition and preferences for their care, and the resources within the LTC home and the acute care system (Bagchus et al., 2024). LTC-to-hospital transitions can present distinct challenges—including potential mismatches between resident needs, LTC and hospital protocols, and disruptions in continuity of care—further heightening the complexity of transfer decisions (Abraham & Menec, 2016; Edgman-Levitan & Schoenbaum, 2021). Previous studies have found that LTC-to-hospital transitions are common worldwide, with reports from Canada indicating that up to half of residents are transferred to an ED within their first year in LTC (Gruneir et al., 2016). While injuries due to fall and infections are a common trigger of LTC-to-hospital transfers, notably, several recent studies have estimated that approximately 40% of ED transfers for LTC residents can potentially be avoided through treatment within the LTC homes by registered practice nurses, registered nurses, and physicians (Canadian Institute for Health Information, 2014; Marincowitz et al., 2022). Unnecessary transfers not only cause emotional distress but also increase the risk of hospital-acquired conditions such as clostridium difficile infections, pneumonia, delirium, and pressure ulcers (Cristina et al., 2021). These risks underscore the importance of the development and implementation of interventions to both effectively reduce unnecessary transitions from LTC-to-hospital and minimize complications associated with the transfer.

Decision support tools are an emerging field, which can serve as an avenue to facilitate the decision-making process for LTC resident transfers to hospital by providing decision-making guidelines and structured frameworks (Lapp et al., 2022). These decision-making tools come in a wide array of forms, including educational booklets, checklists, and multidisciplinary programs (GUIDES expert panel et al., 2018). These tools have the potential to foster clear, empathetic, and informed conversations between residents, care partners, and staff ensuring that the preferences and values of residents and care partners are respected and central to transfer decisions. Multiple recent reviews have been published outlining clinical decision-making tools in LTC; however, current literature evaluating the efficacy of these decision-making tools remains limited (Bavelaar et al., 2023; Kane et al., 2017; Mitchell et al., 2018). Despite the recognized benefits of decision-making tools in LTC, their application is fragmented, with significant variation in efficacy, quality, and availability across LTC homes globally (Lapp et al., 2022). Furthermore, there is a lack of comprehensive reviews systematically mapping existing tools designed specifically for residents and care partners (Lapp et al., 2022). Our review addresses these gaps by providing a systematic analysis of the purpose, implementation, and outcomes of decision-making tools for LTC-to-hospital transfers. Notably, to address these gaps, we conducted a scoping review to systematically identify and map the existing decision-making tools for LTC-to-hospital transfers, with a focus on describing their implementation and reported outcomes to clarify the current landscape and highlight areas for improvement. This knowledge will support the development of more effective and resident-centered tools to enhance decision-making and care quality in LTC settings.

## Methods

### Data Sources and Searches

Our scoping review protocol was registered on Open Science Framework prior to completing abstract screening (Bai et al., 2024). This scoping review followed the methodological framework originally outlined by Arskey and O’Malley and further refined by Levac et al. (Arksey & O’Malley, 2005; Levac et al., 2010). We performed a systematic search of the electronic databases including MEDLINE (from 1946 to present), EMBASE (from 1947 to present), and CENTRAL. The search strategy was developed by the research team with the assistance of a research librarian and was constructed in MEDLINE before subsequently being adapted for use in the EMBASE and CENTRAL databases (Supplemental Table 1). In addition, the primary author manually searched the reference lists of all publications that were included in the scoping review. We searched the electronic databases for subject headings and keywords encompassing the following concepts: (1) decision-making tools; (2) long-term care homes; (3) hospitalization; and (4) resident transfers. The search was limited to the adult population (aged 18 years and older). We did not exclude studies based on the length of LTC residency, as both short-term and long-term residents were eligible for inclusion. The reporting of this scoping review was in compliance with the Preferred Reporting Items for Systematic Reviews and Meta-Analyses Extension for Scoping Reviews (PRISMA-Scr) guidelines (Tricco et al., 2018).

### Inclusion and Exclusion Criteria

We included original studies that evaluated a decision support tool intended to support decisions about transfer to hospital (Table 1). Decision support tools were defined as any tool that aided in the decision-making process by providing structured guidance, standardized protocols, or evidence-based recommendations to assess the necessity of hospital transfers. No year or language restrictions were imposed. We excluded studies that described the development of the decision support tool, rather than evaluated the decision tool.

**Table 1.** Inclusion and exclusion criteria for articles based on study population, decision support tool intervention, study outcomes, and study design

Variable	Inclusion criteria	Exclusion criteria
Study population	Residents and caregivers to residents of long-term care homes, including residential care homes, assisted living homes, and nursing homes	Populations outside the long-term care setting (e.g., community-dwelling elderly and hospital inpatients)
		Studies focusing solely on pediatric or non-geriatric populations
Decision support tool intervention	Studies evaluating decision-making tools specifically designed for transfers decisions from long-term care homes to hospitals	Studies only describing the decision-making tool or focusing on general decision-making tools not specific to transfer decisions
Study outcomes	Studies reporting on outcomes related to the effectiveness of decision-making tools and conversation guides (e.g., measures of communication quality, hospitalization rates, decision conflict, decision satisfaction, resident and family satisfaction, and other transfer outcomes)	Studies without reported outcomes associated with the tool implementation
Study design	Randomize controlled trials, non-randomized trials, observational studies (i.e., cohort, cross-sectional, and case-control), qualitative studies, and mixed-methods studies	Systematic reviews, opinion articles, multimedia files (e.g., online videos and podcasts), case studies, case series, ecological study designs, narrative reviews, editorials, commentaries, and review articles

### Study Selection and Description of Included Studies

Two authors (JB and AM) independently conducted the title and abstract screening for study eligibility using Covidence. Following title and abstract screening, full texts were retrieved then reviewed for inclusion in duplicate (JB and AM). Disagreement concerning inclusion was resolved through consensus meetings. A pre-established data extraction sheet was used to extract data from the included studies. We extracted data of the included studies consisting of variables related to (1) study characteristics, (2) patient demographics, (3) decision support tool details, and (4) study outcomes (Table 2). Specifically, the data extracted relating to study characteristics consisted of the first author of the study, publication year, study title, country the study was conducted in, study design, and the study setting. The patient demographics data included total sample size, attrition rate, sex, age, and patient ethnicity. The data regarding decision support tool details consisted of mode of tool intervention, description of the tool, author and year that tool was developed, purpose of the tool, intended tool users, tool user development (i.e., involvement of residents or care partners in the development of the tool), tool engagement strategy (i.e., the methods and timing of user involvement), and tool implementation into clinical flow (e.g., team training and consistency of application). Lastly, the study outcomes consisted of hospitalization, quality of communication, and additional reported measures.

**Table 2.** Characteristics of included studies

Authors	Year	Total sample size	Study design	Study setting	Mode of intervention	Description of intervention	Results
Bavelaar et al.	2023	88	Pretest-posttest	2 nursing homes in each of Canada, Czech republic, Ireland, Italy, the Netherlands, and the United Kingdom	Educational booklet	Educational booklet for caregivers and a structured family care conference with a trained facilitator to discuss the future care of residents	Reduction in hospitalizations by 62.5% (16 vs. 6) after tool implementation. Improvement in quality of communication by 9.70% (33 vs. 36.2) using the family perceptions of care scale (*p* < 0.05). Decreased decisional conflict was observed using the decisional conflict scale (32.2 vs 22.5; *p* < 0.05). Satisfaction with care (The family perceptions of care scale) was greatly increased (132.5 vs 82.2; *p* < 0.05). The number of documented advance decisions to refuse treatment (e.g., do-not-intubate) was also higher (*p* < 0.05) after the intervention (32%) compared with before (24%)
Blackburn et al.	2020	4320	Pretest-posttest	19 nursing facilities in Indiana	Multidisciplinary program	The optimizing patient transfers, impacting medical quality, and improving symptoms: Transforming institutional care (OPTIMISTIC) program employs full-time registered nurses to work with staff at each facility to assess and address changes in resident condition and quality improvement efforts. Additionally, OPTIMISTIC nurse practitioners provide in-person evaluations, and management of residents with acute condition changes. Evidence-based processes implemented under OPTIMISTIC include coordination of care through collaborative care reviews, respecting choices advanced steps advance care planning facilitation, and the use of tools from interventions to reduce acute care transfers	12.25% decrease in probability of hospitalization (0.49 vs 0.43; *p* < 0.001) after tool implementation
Einterz et al.	2014	18	Pretest-posttest	2 nursing homes in North Carolina	Decision aid video	Goals of care decision aid video viewed by the surrogate decision-maker, and a structured care plan meeting between the surrogate decision-maker and interdisciplinary nursing team. The decision aid video content includes information about advanced dementia, role of the surrogate decision-maker, treatment goals, and treatment options consistent with each goal. Provided in video and print formats, it uses patient stories, balanced presentation, and simple language to enable comprehension at an 8th grade educational level. The decision aid encouraged further discussion with healthcare providers and included a printed guide to assist in this discussion	Improved quality of communication scores (6.1 vs 6.8, *p* = 0.01) and improved concordance on primary goal of care with nursing home team (50% vs 78%, *p* = 0.003). The number of palliative care domains addressed in the care plan increased (1.8 vs 4.3, *p* < 0.001). Surrogates also showed a trend indicating that more of them were “very involved” with decision-making about goals of care after the intervention (56% vs 83%). 89% of the surrogate decision-makers (SDMs) thought the decision aid was relevant to their needs. After viewing the video decision aid, SDMs increased the number of correct responses on knowledge-based questions (12.5 vs 14.2; *p* < 0.001)
Hanson et al.	2016a	302	RCT	22 nursing homes in the United States	Decision aid video	18-minute goals of care video decision aid (providing information on dementia, goals of prolonging life, supporting function, how to prioritize goals, improve comfort, and treatments consistent with each goal) and a structured discussion with the nursing home care team	There was a 50.75% lower hospitalization count (33 vs. 67; *p* = 0.02) and a 7.14% higher report of quality of communication (6 vs. 5.6; *p* = 0.05) for family decision-makers (assessed using a goals of care questionnaire) in the intervention group compared to the control group
Hanson et al.	2016b	151	RCT	22 nursing homes in North Carolina	Decision aid video	The family decision-makers in the intervention group received a two-component intervention: a Video decision aid about GOC care choices in advanced dementia and a structured nursing home care plan meeting to address GOC. Three goals are discussed in the 20-minute decision aid: prolonging life, supporting function, and improving comfort. Each goal is described along with treatment options consistent with prioritizing that goal. Further, each goal was illustrated with a story of a person who used the goal to guide treatment choices. After viewing the decision aid during the enrollment interview, family decision-makers were given a copy of the decision aid and a print discussion guide to use with healthcare providers. The discussion guide provided stepwise guidance in how to use the decision aid content in shared decision-making	Goals of care were discussed in 123 (92%) meetings. Choice of the primary goal of care was made in 127 (95%) meetings. The treatment plan for 130 (97%) patients was confirmed or changed after the discussion. Although investigators encouraged GOC discussions soon after the video decision aid component, 94 (69%) of the discussions were completed within 3 months. 127 (84%) of the discussions occurred within 6 months
Ingber et al.	2017	NR	Mixed-methods study	143 nursing homes within seven states	Multidisciplinary program	The initiative consisted of seven enhanced care and coordination provider models of interventions in seven states aimed at improving the overall health and health care of participating long-stay residents of nursing facilities. Although CMS provided guidelines for intervention design and required that each model contain certain key elements, ECCPs had the flexibility to select and implement specific interventions. The required elements included hiring on-site facility staff to focus on improvements related to avoidable hospitalizations, such as medication management and improving communication and coordination among nursing facility staff members, residents’ primary care providers and specialists, pharmacies, and hospitals. To improve care processes and communication among providers, all ECCPs chose to use a nursing home quality improvement program known as interventions to reduce acute care transfers (INTERACT). ECCPs used one or more of the following INTERACT tools: The stop and watch early warning tool assists staff members who witness a change in a resident’s condition to notify nurses. “Care paths,” which are symptom specific, provide decision support tools for nurses. The situation, background, assessment, and recommendation (SBAR) tool provides a template for nurses to use in assessing a resident’s condition before notifying a physician of a change in that condition. The nursing home–Hospital transfer form organizes resident records to simplify communication between staff at the two institutions involved in the transfer. Lastly, the quality improvement tool for review of acute care transfers facilitates review of acutely ill residents transferred to the hospital by facility staff members, often with the goal of determining whether the hospitalization was avoidable	Reduction in hospitalizations each year because of the initiative. Reduction in medicare expenditure each year because of the initiative
Kane et al.	2017	36,717	RCT	85 nursing homes	Multidisciplinary program	The study team provided training and support for implementing the interventions to reduce acute care transfers (INTERACT) program, which includes a set of tools that address the key factors leading to avoidable hospital admissions and ED visits among NH residents. The INTERACT program includes over 30 clinical and educational tools, that include quality improvement tools to measure and track hospitalization outcomes, tools for improving documentation and communication within the NH and between the NH and the hospital, decision support tools including 10 care paths for conditions commonly associated with potentially avoidable hospitalizations, and advance care planning tools to encourage goals of care discussions and reduce hospitalizations in people with end-stage illness among whom the risks and discomforts of hospital care often outweigh the benefits	There was a reported reduction of 0.13 per 1000 resident days in hospitalizations. Specifically, they found a reduction of 0.37 per 1000 resident days in hospitalizations during the first 30 days of admission, and a reduction of 0.09 per 1000 resident days in hospitalizations during periods more than 30 days after nursing home admission when compared to control nursing homes. 30-day readmission rates reduced by 0.01. Emergency departments visit without admission increased by 0.02 per 1000 resident days. Reduction in potentially avoidable hospitalizations overall (−0.18 per 1000 resident days; *p* = 0.01)
Mitchell et al.	2018	402	RCT	64 nursing homes in Boston	Decision aid video	A 12-minute ACP video for proxies with written communication of their preferred level of care (comfort, basic, or intensive) to the primary care team	There was a 14.41% lower hospitalization rate (9.5% vs. 11.1%) in the intervention group compared to the control group. Do not hospitalize directives did not differ between the intervention and control group (63% in both arms). Preferences for comfort care, directives to withhold intravenous hydration, and burdensome treatments did not differ between both arms. Residents in the intervention vs control facilities were more likely to have directives for no-tube-feeding (70.10% vs 61.90%; *p* < 0.05). Residents in the intervention vs control facilities were more likely to have documented goals of care discussions (16.10% vs 7.90%; *p* < 0.05). When comfort care was preferred, residents in the intervention arm were more likely to have both do not hospitalize and no-tube-feeding directives (72.20% vs 52.80%; p < 0.001). The rate of burdensome treatments (i.e., hospital transfers, feeding tube insertions, or parenteral therapy) per 1000 resident days did not differ significantly between both arms (1.23 control vs 1.42 intervention arm)
Molloy et al.	2000	1292	RCT	6 nursing homes in Ontario	Advance directive program	The let Me decide advance directive program included educating staff in local hospitals and nursing homes, residents, and families about advance directives and offering instructional and proxy components, providing a range of health care choices for life-threatening illnesses, cardiac arrests, and feeding	There was a 7.66% (197 vs. 183) increase in hospitalization rate in the intervention nursing home compared to the control nursing home at a 12-month assessment period. There was a 50.69% (143 vs. 290) decrease in hospitalization rate in the intervention nursing home compared to the control nursing home at an 18-month assessment period. Satisfaction was not significantly different in intervention and control nursing homes. Intervention nursing homes reported less resource use than control nursing homes (average total cost per patient $3490 vs $5239). The proportion of deaths in intervention (24%) and control (28%) nursing homes were similar
Ouslander et al.	2011	NR	Quality Improvement Study	25 nursing homes in 3 US states (Florida, Massachusetts, and New York)	Multidisciplinary Program	A refined Interventions to Reduce Acute Care Transfers (INTERACT) program was implemented, which consisted of a set of evidence-based clinical practice tools and strategies. The tool incorporated leadership education, facility champion, nursing home education, collaborative telephone conference calls, communication tools, early warning tools, situation-background-assessment recommendation communication tools and progress notes, change in condition file cards, resident transfer forms, acute care transfer checklists, advance care planning tools, and acute transfer reviews (quality improvement analysis)	There was a 16.80% reduction in hospitalization rates after the intervention was implemented (3.99 vs. 3.32 per 1000 resident days; *p* = 0.02)
Popejoy et al.	2019	5168	Cross-sectional	16 nursing homes in Midwestern United States	Multidisciplinary Program	Nurses used various strategies to influence hospital transfer rates including performing a root cause analysis on all transfers using a slightly adapted INTERACT Quality improvement Acute Care Transfers (ACTs) tool, v.3.0. Nurses completed ACTs for each transfer using a combination of chart review and nurse interviews. ACT quality improvement procedures included (1) monthly review of all ACTs by a team composed of the APRN, project coordinator, and nurse supervisor; (2) identification of resident and NH factors contributing to the transfer; and (3) agreement by the team on the question “Was the transfer potentially preventable (avoidable)?” using the quality improvement technique “FiveWhy’s	A total of 54% of hospital transfers were identified as avoidable. Quality improvement opportunities related to avoidable transfers were earlier detection of new signs/symptoms (OR = 2.35; *p* < 0.001). Improvement in discussions of resident/family preference (OR = 2.12; *p* < 0.001). Improvement in advance directive/hospice care planning (OR = 2.25; *p* = 0.003. Better communication about condition (OR = 4.93; *p* < 0.001). Condition identified as could have been managed in the nursing (OR = 16.63; *p* < 0.001)
Tappen et al.	2020	192	RCT	15 long-term care facilities	Educational Booklet	The decision aid “Go to the Hospital or Stay Here?” was developed from data obtained in a previous study by Tappen et al. The guide includes indications of when a decision to transfer to acute care or remain in the nursing home setting is appropriate. The Guide also contains a list of assessments and interventions that can be provided in most nursing homes to address the evidence of limited awareness of the capabilities of long-term care facilities. A frequently asked questions section addressing assumptions and misunderstandings evident in participant responses and frequently expressed concerns section about personal belongings and how family members are informed if a transfer becomes necessary were also addressed within the Guide	There was an increase in hospitalizations by 60.00% (5 hospitalizations in the control group and 8 in the intervention group) in the intervention group compared to the control group. Use of the Guide (intervention group) was found to increase residents’ and family members’ knowledge between pretest and post-test scores in the assigned knowledge test (p = 0.006). The intervention was found to significantly decrease decisional conflict compared to the control group (8.95 vs 15.73; p = 0.001). The intervention group did not increase decisional preparation. There were no significant differences between the intervention and control groups on expressed preference to remain in the nursing home or transfer to acute care. Residents and families rated the Guide as very helpful
Tsai et al.	2018	583	Pretest-Posttest	NR	Checklist	The nursing home to emergency room (NHERT) checklist consisted of four main parts: (i) demographic data of the NH resident: name, date of birth, gender, past medical history, past history of medical treatment, allergy, current medications, tube use status and code status; (ii) critical data for NH-to-ER transfer: symptom or reason for transfer and health status at transfer; (iii) contact information: information regard key person, agency information; and (iv) critical data for ER to NH transfer: ER diagnosis, ER tests, follow-up care, recommended medications	Use of the nursing home to emergency room transfer checklist resulted in a 30-day readmission rate of 13.4%, which was lower than the baseline rate of 15.9%
Vogelsmeier et al.	2021	NR	Cohort	16 nursing homes in Midwestern United States	Multidisciplinary Program	An advanced practice registered nurses (APRN) was embedded full-time as essential staff within each NH and focused on basics of care delivery, early illness detection, acute illness management, medication reviews to reduce polypharmacy, and systems change through quality improvement (QI) efforts. The Intervention to Reduce Acute Care Transfers (INTERACT) program was implemented in each home. Monthly feedback reports were provided on facility transfer rates to use for QI efforts. Health information technology (HIT) was implemented, specifically health information exchange (HIE), to facilitate communication between NH staff and providers	There was a 23.79% (2.48 hospital transfers per 1000 resident days vs. 1.89 hospital transfers per 1000 resident days) decrease in hospitalization after the intervention was implemented at a 4-year assessment period. There was a 19.76% (2.48 hospital transfers per 1000 resident days vs. 1.99 hospital transfers per 1000 resident days) decrease in hospitalization after the intervention was implemented at a 5-year assessment period
Zuniga et al.	2021	942	NRCT	11 nursing homes in German-speaking Switzerland	Multidisciplinary Program	The INTERCARE model is composed of 6 evidence-based core components with minimal requirements for each and adaptable elements for local tailoring to reduce transfers from the nursing home to hospital. The core components included 1) strengthening of interprofessional collaboration between physicians and nursing home staff through the development of internal structures; 2) an INTERCARE nurse specifically appointed in each nursing home; 3) comprehensive geriatric assessment of residents initiated by INTERCARE nurses when a change in condition was observed; 4) the use of evidence-based tools from the INTERACT program, including STOP and WATCH, ISBAR, and a reflection tool to analyze reasons for unplanned transfers; 5) advanced-care planning to help nursing homes initiate sensitive discussions and document residents’ wishes; and 6) data-driven quality improvement to identify areas for improvement (e.g., hospital transfers)	Hospitalization rates were assessed at 4 timepoints: 3 months, 6 months, 9 months, and 12 months. The baseline hospitalization rate was 0.84 per 1,000 resident days. Respectively, there was an increase by 1.19% (0.85 per 1000 resident days), decrease by 23.81% (0.64 per 1000 resident days), decrease by 5.95% (0.79 per 1000 resident days), and decrease by 50% (0.42 per 1000 resident days). During the 3 month-baseline and 18-month intervention study periods, 367 hospital transfers occurred, of which 303 transfers (82.6%) were unplanned (primary outcome) and 64 transfers (17.4%) planned

### Analysis

A descriptive analytical approach was used to summarize and map the findings of the included studies. Studies were categorized into domains based on the format and intended function of the decision support tool. Key characteristics such as study design, intervention components, implementation strategies, and reported outcomes were summarized narratively. This mapping allowed for a high-level comparison of intervention types and identification of common implementation practices and reported outcomes. No critical appraisal or risk of bias assessment was conducted, in keeping with scoping review methodology.

## Results

### Identification of Studies

Electronic database searches identified a total of 1383 papers (Figure 1). After the removal of 89 duplicates, the remaining 1303 studies underwent title and abstract screening, and 37 of these papers met the inclusion criteria and were retrieved for full-text review. Of these 46 full-text articles, 31 studies were excluded for the following reasons: 20 studies did not implement a decision support tool into practice, eight did not evaluate any outcomes as a result of the decision support tool intervention, one did not have LTC residents as its intended population, and one did not have an appropriate study design. As a result, the full-text screening yielded six studies to be included in the scoping review; an additional nine were identified through the manual review of the reference lists of the included studies. Ultimately, 15 studies were included in the scoping review.

**Figure 1.**
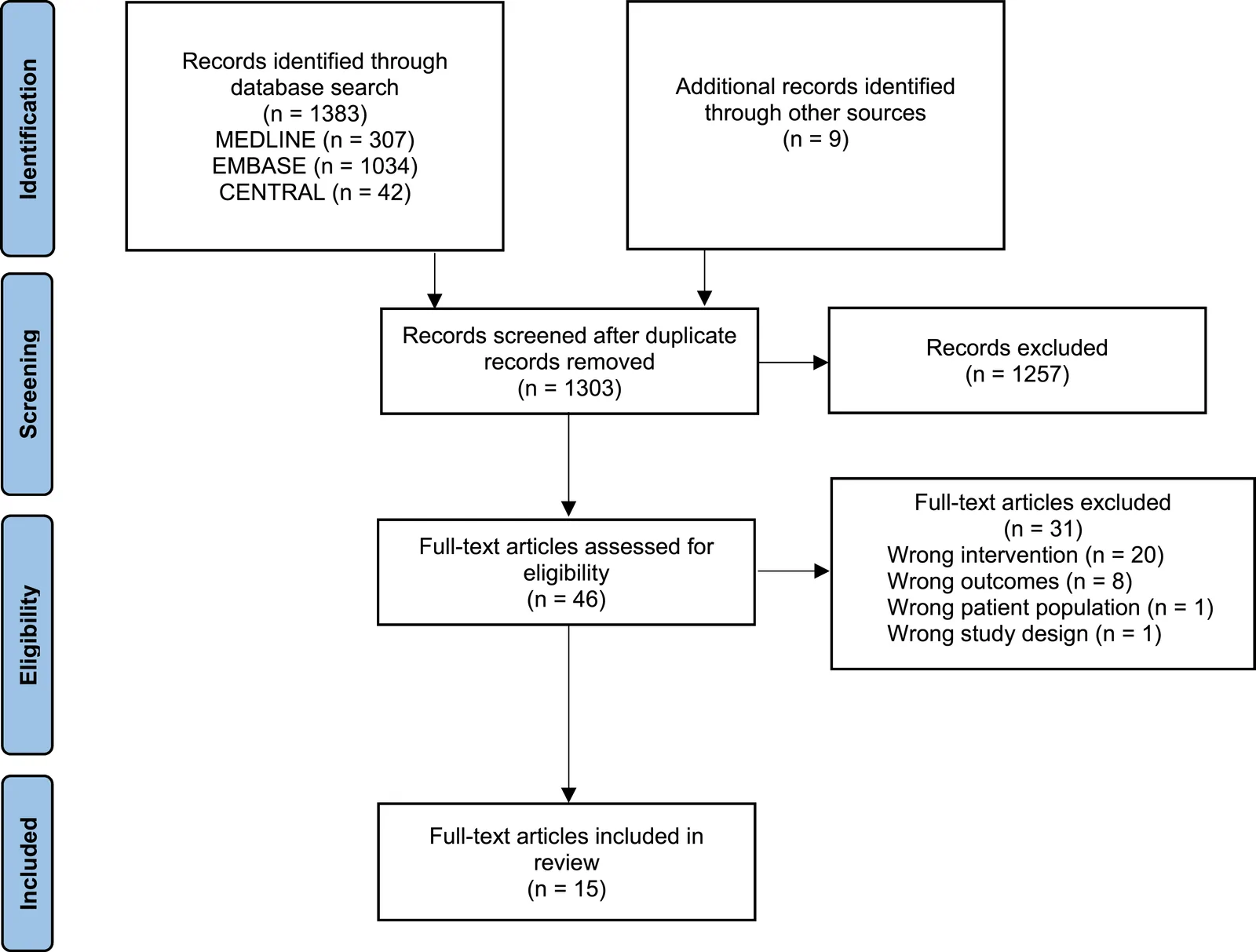
Flow diagram of literature screening using the preferred reporting items for systematic reviews and meta-analyses (PRISMA) guidelines. Figure adapted from https://prisma-statement.org

### Description of Studies

The 15 included studies were published between 2000 and 2023, with the majority published after 2016 (n = 12) and none published in 2024. Of the included studies, most were conducted in the United States (n = 11), and the remaining studies were conducted in Canada (n = 1), Switzerland (n = 1), Taiwan (n = 1), and the Netherlands (n = 1). Notably, over one-third (n = 6) of the included studies were randomized controlled trials. The sample size varied greatly ranging from 18 to 36,717 LTC residents. The decision-making tools included educational booklets, checklists, multifaceted programs that incorporated various communication tools, quality improvement tools, and communication forms. Most interventions (n = 7) were multidisciplinary and incorporated several components such as advance care planning, caregiver education, and patient communication strategies. Thirteen studies examined hospitalization outcomes.

The 15 included studies were then divided into five domains—to systematically group information based on shared characteristics, allowing for a structured mapping of different decision-making support approaches—based on the format of the decision support tool intervention: (1) Education Booklet, (2) Decision Aid Video, (3) Multidisciplinary Program, (4) Advance Directive Program, and (5) Checklists (Casimir et al., 2022). The Educational Booklet domain encompassed studies that developed booklets consisting of essential information to prepare residents for transfer decisions, involving them in decision-making, and setting their expectations for hospital transfers from LTC. The Decision Aid Video domain included studies that implemented various video formats to inform residents about their involvement in hospital transfer decisions and goals of care. The Multidisciplinary Program consisted of studies that used multiple tools to facilitate decision-making for hospital transfers from LTC. The Advance Directive Program domain involved study interventions that used advance directives (e.g., formal documentation of residents’ preferences for medical treatment and end-of-life care) to facilitate transfer decisions and enhance resident-centered care. Lastly, the Checklist domain included studies that used resident checklists as the mode of intervention to improve communication and care transitions between the hospital and LTC home.

### Domain 1: Educational Booklets for Transfer Decision-Making

Of the 15 included studies, two developed educational booklets to facilitate decision-making and hospital transfers from LTC by enhancing resident and care partner preparedness (e.g., by outlining the potential benefits and risks of transfer, expected outcomes, alternatives to hospital transfer, resident preferences and advance directives, contact information for healthcare providers, and signs or symptoms warranting a transfer) (Bavelaar et al., 2023; Tappen et al., 2020). Both studies employed the tool at the start of the study, distributing them to care partners or residents to equip them with information necessary for making informed transfer decisions.

Bavelaar et al. developed an educational booklet discussing the trajectory of dementia, possible symptoms and complications, and the importance of shared decision-making, in addition to a subsequent family/friend care conference with a trained facilitator to further discuss the future care of the LTC resident living with dementia. Specifically, the educational booklet was given at the start of the study and aimed to inform the care partners about end-of-life care options for the residents living with dementia and to support them in their decision-making process (Bavelaar et al., 2023). As a result of this intervention, Bavelaar et al. reported that there was a 62.5% reduction in hospitalization counts after the tool was implemented. Moreover, they found that the quality of communication improved by 9.7% postintervention using the Family Perceptions of Care Scale (Bavelaar et al., 2023).

Tappen et al. developed an educational booklet titled “Go to the Hospital or Stay Here” that includes indications of when a decision to transfer to acute care or remain in the nursing home setting is appropriate. It provided information on what to expect from the transfer (e.g., hospital processes), outlined the importance of advance directives, and also offered the testimonials of other residents who had experienced this transfer, describing their personal experience (Tappen et al., 2020). Importantly, they found that the implementation of the tool substantially decreased decisional conflict and increased resident knowledge regarding the transfer. Additionally, they reported a 60.0% increase in hospital transfers in the intervention group. This increase, however, may have been attributed to the improved knowledge and reduced decision conflict among the family members and residents, which could have led to a greater willingness to seek hospital care when appropriate.

### Domain 2: Decision Aid Videos for Goals of Care Discussions

Four studies used a decision aid video as their intervention to improve hospital transfers and decision-making among residents (Einterz et al., 2014; Hanson et al., 2016, 2017; Mitchell et al., 2018). The decision aid videos varied in content; however, all studies included information about how to discuss patient health goals with their care team to improve communication between LTC home staff and residents.

Hanson et al. implemented an 18-minute goals of care decision aid video that provided information on dementia (e.g., symptomology), goals of improving comfort and prolonging life as well as treatment options for each goal. In addition, the intervention incorporated a structured discussion with the LTC staff. The decision aid aimed to reduce hospital transfers and improve end-of-life communication between decision-makers and clinicians. Notably, there was a 50.8% reduction in hospital transfers and a 7.1% increase in quality of communication as a result of the tool using a validated quality of communication tool (Hanson et al., 2017).

Mitchell et al. used a 12-minute advance care planning video, which aimed to improve resident and care partner documentation of advance directives, facilitate goals of care discussions, and record level of care preferences among LTC residents living with dementia (e.g., wishes for resuscitation and intubation) (Mitchell et al., 2018). They reported a 14.4% reduction in hospitalization rates. In addition, residents in the intervention group were reported to had a higher likelihood of having documented goals of care discussions (Mitchell et al., 2018).

Next, Einterz et al. had an intervention that included an 18-minute goal of care decision aid video in addition to a structured care plan meeting with the interdisciplinary nursing team. The decision aid was viewed by residents’ substitute decision-makers and included information regarding dementia, their roles and responsibilities as substitute decision-makers, and treatment options to meet the primary goals of longevity, function, or comfort. The aim of the intervention was to promote the choice of medical care to prolong life and enhance resident comfort. Additionally, they aimed to improve the quality of communication to better support substitute decision-making. Einterz et al. reported an 11.5% increase in quality of communication after implementation. Furthermore, they reported an improved concordance on primary goals of care with the LTC home staff, and increased surrogate decision-maker self-reported involvement in the transfer (Einterz et al., 2014).

Lastly, a second study by Hanson et al. (2016), used a 20-minute goals of care video decision aid and a structured, planned meeting between the family decision-maker and the LTC home staff. The decision aid aimed to improve the quality of communication and facilitate decision-making among residents. They reported that the residents’ goals of care were discussed in 92.0% of the meetings and that 97.0% of residents confirmed pre-arranged treatment plans after use of the tool (Hanson et al., 2016).

### Domain 3: Multidisciplinary Programs for Reducing Hospital Transfers

Seven studies implemented a multidisciplinary program, which consisted of a variety of tools to support decision-making for LTC-to-hospital transfers. Specifically, all seven multidisciplinary programs used the Interventions to Reduce Acute Care Transfers (INTERACT) program (Abraham & Menec, 2016; Bavelaar et al., 2023; Blackburn et al., 2020; Einterz et al., 2014; Popejoy et al., 2019; Tsai & Tsai, 2018; Vogelsmeier et al., 2021), which encompasses a set of communication and planning tools. The tools of all 7 studies aimed to identify acute changes in resident health conditions and address primary factors leading to avoidable resident hospital admissions including insufficient advance care planning, better decision support to respond to changes in condition without requiring hospital transfers, and fill gaps in communication between healthcare providers and LTC staff. In brief, the INTERACT program, which was included in the intervention of each of the 7 studies, entails leadership education, an early warning tool, a situation-background-assessment recommendation communication tool, a resident transfer form, an advance care planning tracking tool, and transfer checklists.

Six of the seven studies that used the INTERACT program reported on hospitalization outcomes finding a reduction in hospitalization with the INTERACT program (Blackburn et al., 2020; Ingber et al., 2017; Kane et al., 2017; Ouslander et al., 2011; Vogelsmeier et al., 2021; Zúñiga et al., 2022). Zuniga et al. reported the hospitalization rates at 3-month, 6-month, 9-month, and 12-month intervals finding a 1.2% increase, 23.8% decrease, 6.0% decrease, and 50.0% decrease from baseline, respectively (Zúñiga et al., 2022). Kane et al. also reported hospitalization outcomes at various time points: less than 31 days following admission into LTC and greater than 30 days. The authors reported a reduction in hospitalizations of 0.13 per 1000 resident days in the intervention group compared to the control nursing homes. Specifically, they reported a reduction of 0.37 per 1000 resident days during the first 30 days of nursing home admission, and 0.09 per 1000 resident days after the first 30 days of nursing home admission (Kane et al., 2017). Moreover, Vogelsmeier examined hospitalization outcomes at the 4-year and 5-year time points and found a 23.8% and 19.8% decrease, respectively. Ouslander et al. described a reduction of 16.8% in hospitalization rate as a result of the INTERACT intervention (Ouslander et al., 2011). Next, although Ingber et al. did not outline a quantitative measure of hospitalization rate changes, they observed a reduction in hospitalizations each year after the INTERACT initiative was implemented (Ingber et al., 2017). Notably, one of the seven studies that implemented a multidisciplinary decision-making intervention utilized both the INTERACT program and the Optimizing Patient Transfers, Impacting Medical Quality, and Improving Symptoms: Transforming Institutional Care (OPTIMISTIC) program. This program employs full-time nurses to closely work with LTC staff to assess and address acute changes in resident health conditions and foster quality improvement efforts such as advance care planning, proactive chronic disease management, and improving communication between LTC staff and families. Additionally, the OPTIMISTIC program includes coordination of care through collaborative care reviews and advance care planning facilitation. Blackburn et al. reported a 12.3% decrease in the probability of hospitalization after the OPTIMISTIC and INTERACT programs were introduced to the LTC homes. Lastly, Popejoy et al. utilized the INTERACT program as the primary decision-making tool; however, instead of reporting changes in hospitalization rates, they investigated transfer preventability and identified 54.0% of hospital transfers as preventable (Popejoy et al., 2019). They also found improved communication about the condition, earlier completion of advanced directives, and earlier discussions of resident care preferences as a result of the tool (Popejoy et al., 2019).

### Domain 4: Advance Directive Program for Resident-Centered Care

One study introduced a tool design consisting of an advance directive program to support transfer decisions and improve resident-centered care (Molloy et al., 2000). Molloy et al. formulated a program to educate residents, care partners, and LTC and hospital staff about advance directives by providing instructional and proxy components (i.e., decision-making frameworks for palliative care, feeding preferences, and resuscitation) while outlining treatment options for various acute changes in health condition (Molloy et al., 2000). The authors reported a 7.7% increase in transfers at the 12-month mark, and a 50.7% reduction at 18-months compared to the pre-period. Moreover, homes using the intervention had lower cost per patient (average total cost per patient of $3490 vs $5239) compared to control LTC homes (Molloy et al., 2000). Additionally, the proportion of deaths in the intervention (28%) and control (24%) nursing homes were similar.

### Domain 5: Communication Checklists for Improving Care Transitions

One study used a checklist to improve communication and care transitions (Tsai & Tsai, 2018). Tsai et al. developed a LTC home to Emergency Room (NHERT) checklist that included four key domains: (1) demographic information of the LTC home resident, (2) contact information relevant to the resident, (3) critical health information for the transfer including the symptoms or reason for transfer as described by a nurse, and (4) medication recommendations and follow-up care preferences. The checklist aimed to improve care transitions by fostering better communication between emergency departments and LTC homes and ensuring that critical information is preserved throughout the transition. Tsai et al. found the NHERT checklist reduced 30-day readmission rates by 2.5%.

## Discussion

This scoping review demonstrated that decision-making tools facilitate LTC-to-hospital transfers and reducing hospitalization associated outcomes. The integrated findings of the 15 included studies offer a comprehensive understanding of the complexity of transfers from LTC homes to hospital and highlight the variability in intervention methods as outlined by the categorized domains. Specifically, this scoping review identified 15 studies of interventions that improved various aspects associated with the LTC-to-hospital transfer process, which included six randomized controlled trials, four pre-/post-intervention study, one non-randomized controlled trials, one cohort study, one cross-sectional study, one mixed-methods study, and one quality improvement study. While the tools varied in format and variety, many studies reported improvements in communication, documentation, and hospitalization outcomes. These findings reflect a growing recognition of the importance of structured decision support in LTC, particularly in contexts where hospital transfers can have significant consequences for cognitively impaired residents. Additionally, although the current research highlights the potential of various interventions to aid in facilitating transfers from LTC-to-hospital, but it does not offer compelling evidence for a single definitive solution.

Prior research has reported that up to 40% of LTC-to-hospital transfers may be avoidable, with various contributing factors (Marincowitz et al., 2022). The tools identified in this review address these challenges in different ways, from educational booklets and video aids that facilitate shared decision-making, to multidisciplinary programs that embed systemic quality improvement strategies. Although each study implemented different decision aids to facilitate the transition from LTC-to-hospital, several important similarities and distinctions emerged. The way that tools are implemented and how intended users engage with them is critical in ensuring the efficacy of a decision aid intervention. Notably, the majority of studies that outlined their tool engagement strategy (9 out of 13) employed an ongoing engagement approach rather than a one-time interaction throughout the study and generally involved the inclusion of refresher sessions and follow-up webinars. Additionally, 11 of the 15 included studies targeted nurses or nursing home staff, while two focused on family caregivers, one on the primary care team, and one on the resident or their family members.

### Bridging Gaps in Tool Applications

This review revealed significant variability in both the tools themselves and their outcomes. For instance, educational booklets in two studies resulted in improved communication, but the impact on hospitalization rates was mixed, with one study reporting reductions while the other saw increases. Similarly, multidisciplinary programs like INTERACT consistently reduced hospitalizations, yet the degree of reduction varied widely across studies. These discrepancies suggest that the context in which these tools are implemented—such as the care setting, the engagement strategies, and the specific health needs of the residents—plays a crucial role in determining their effectiveness.

The variability noted in this review’s findings suggests the need for standardized, evidence-based tools that can be applied consistently in LTC settings. Research by Ouslander et al. (2014) highlighted how structured frameworks can reduce avoidable transfers (Ouslander et al., 2014). However, even this program, widely regarded as a best practice, was not universally effective, indicating that even the most well-designed tools may need to be adjusted to fit the specific needs and conditions of each care setting to achieve optimal outcomes. This aligns with findings from implementation science, highlighting that tools often need to be tailored to the specific context in which they are used rather than applied uniformly (Moullin et al., 2020). Contextual factors such as organizational culture, staff training, resource availability, and resident demographics significantly influence the success of implementing decision-making tools in care settings (Choopani et al., 2024). Customization ensures that tools address unique conditions and meet the needs of stakeholders effectively, ultimately improving outcomes (Chen et al., 2023).

Further research is needed to develop adaptable frameworks that allow for customization based on resident demographics, LTC home resources, and regional healthcare policies, while still maintaining core principles that guide effective decision-making.

### Continuous Engagement and Multidisciplinary Collaboration

Our review emphasized the importance of continuous engagement in decision-making tool implementation. Studies that employed ongoing engagement strategies, such as refresher sessions and follow-up webinars, reported better outcomes compared to those relying on one-time interactions. This aligns with findings in other areas of healthcare, where ongoing professional development and reinforcement have been shown to improve adherence to guidelines and protocols (Wensing et al., 1998). Continuous engagement ensures that staff remain familiar with the tools, fosters a culture of ongoing learning, and allows for the iterative refinement of care practices based on evolving resident needs.

Moreover, the success of multidisciplinary programs, particularly those incorporating the INTERACT and OPTIMISTIC models, highlighted the value of collaboration across healthcare disciplines. To this end, these interventions facilitated communication between LTC staff, residents, and external care providers, such as ED teams and primary care physicians. By connecting areas of healthcare that are typically siloed, multidisciplinary programs not only reduced hospitalizations but also enhanced the overall quality of care that residents received. However, a critical gap remains: the limited involvement of residents and their families in the decision-making processes of these tools (Birchley et al., 2016; Wallace, 2015). By including residents and care partners as active participants in developing and testing interventions targeting transfers, the resulting tools would be better aligned with resident preferences (Mitchell et al., 2018).

### Resident-Centered Care and Empowerment

Despite the growing emphasis on resident-centered care in healthcare literature, most tools identified in this review were primarily targeted at healthcare professionals, particularly LTC staff. Only a small number of interventions were designed with a direct focus on engaging residents and their families. This is a significant oversight, given the increasing body of evidence showing that shared decision-making improves both health outcomes and patient satisfaction (Elwyn et al., 2012). Tools that empower residents to make informed choices about their care, particularly those involving critical decisions such as hospital transfers, are essential for promoting autonomy and improving quality of life in LTC populations (Ménard et al., 2024).

While decision aid videos showed promise in improving goal of care discussions and increasing advance directive completion, their limited use suggests a need for broader implementation. Videos that addressed specific clinical scenarios, such as those employed by Hanson et al. (2016), not only facilitated better communication but also provided residents and families with the information they needed to make value-based decisions (Hanson et al., 2016). As the population in LTC continues to grow and diversify, the need for decision-making tools that are culturally sensitive and accessible to residents with varying levels of cognitive function will increase. This will require not only the inclusion of residents in tool co-development but also an improved understanding of the diverse cultural and social factors that influence health-related decision-making.

### Addressing Cultural and Demographic Gaps

One of the most glaring gaps in the literature, as highlighted by this review, is the lack of attention to cultural, geographic, and demographic differences in the development and application of decision-making tools. None of the included studies explicitly examined how factors such as ethnicity, socioeconomic status, or geographic location might influence transfer decisions. While many studies may have lacked the power to conduct subgroup analyses, the lack of attention to these contextual factors represents an important opportunity for future research, especially given the increasing diversity in LTC populations and the growing body of evidence suggesting that such factors can shape healthcare preferences and outcomes (Betancourt et al., 2003). For example, research has shown that cultural factors can influence end-of-life care preferences, with some populations placing greater emphasis on family involvement, while others prioritize individual autonomy (Kwak & Haley, 2005). The absence of culturally tailored decision-making tools may result in care that is misaligned with the values and preferences of residents from minority backgrounds. Developing tools that are culturally sensitive and that accommodate a wide range of health literacy levels will be vital for ensuring equitable care in LTC settings.

### Strengths and Limitations

Although our findings serve as a foundation to inform future research and will facilitate the development of a novel, resident-centered decision-making tool for LTC home to hospital transfers, this review has several limitations. Firstly, there was significant heterogeneity in terms of the study designs, intervention type, and the reporting of assessed outcomes in each included article. As a result, the variability raises difficulty in comparing the results across studies to assess the efficacy of certain tools. Specifically, while hospitalization outcomes were the most consistently reported across the studies, not all studies included this data. Another notable limitation of this review is the absence of consideration of cultural and demographic differences among different LTC homes. Importantly, decisions regarding transitions from LTC-to-hospital are highly dependent on the resident’s values and goals, which can vary greatly based on their culture, location, and individual circumstances (Abraham & Menec, 2016). None of the included studies incorporated factors that considered cultural sensitivity or accessibility, which could be improve both the decision-making process and the LTC home to hospital transfer experience for the resident.

### Implications for Future Research and Practice

The findings from this scoping review provide several important implications for both practice and future research. First, healthcare providers and policymakers should prioritize the development of standardized, evidence-based decision-making tools that can be adapted to local contexts while maintaining core principles that guide effective transfer decisions. Such tools should be designed to engage not only healthcare professionals but also LTC residents and their families, empowering them to participate actively in their care.

Future research should focus on the long-term efficacy of these tools, particularly in terms of sustained reductions in hospitalizations and improvements in resident outcomes such as preparedness for decision-making, patient satisfaction, and communication between residents, families, and healthcare providers. Randomized controlled trials and longitudinal studies can be leveraged in assessing whether these tools deliver lasting benefits or if their effectiveness diminishes over time. Additionally, more research is needed to explore how cultural and demographic factors influence the decision-making process, and how tools can be designed to accommodate diverse resident populations.

Furthermore, given that many included studies were conducted in the post-Affordable Care Act era in the United States, interventions may have been shaped by policy incentives aimed at reducing hospital readmissions. While our review did not limit inclusion based on resident length of stay, future research should explore how policy environments and resident type (short-term vs. long-term) influences the design and effectiveness of decision-making tools.

Finally, there is a need to integrate continuous engagement strategies into the implementation of decision-making tools. The success of ongoing support in the form of refresher sessions and follow-up activities should be built into the development of future interventions to ensure that healthcare professionals, residents, and families have the resources and support needed on an ongoing basis to make informed, value-based decisions about their care.

## Conclusion

This scoping review offers a comprehensive overview of existing decision-making tools for LTC-to-hospital transfers, while also revealing gaps in their application and effectiveness. Importantly, we identified a gap in the explicit cultural adaptation of tools and limited involvement of residents and families in tool development. These findings emphasize the need for standardized yet adaptable decision-making frameworks that can be tailored to diverse LTC populations and implemented consistently across care settings. Future research should prioritize longitudinal evaluations, co-design approaches involving residents and families, and assessments of cultural relevance to ensure equitable, sustainable, and person-centered improvements in the LTC-to-hospital transfer process.

## Supplemental Material


Supplemental material - Assessing Decision-Making Tools for Meaningful Discussions About Transfer Decisions From Long-Term Care to Hospital: A Scoping Review
Supplemental material for Assessing Decision-Making Tools for Meaningful Discussions About Transfer Decisions From Long-Term Care to Hospital: A Scoping Review by Jia Qi Adam Bai, Alixe Ménard, Yamini Singh, Daniel Kobewka, and Krystal Kehoe MacLeod in Journal of Applied Gerontology.

## Data Availability

The data underlying this article are available in the article and in its online supplementary material.*
