# Supplementary material for: Assessing Decision-Making Tools for Meaningful Discussions About Transfer Decisions From Long-Term Care to Hospital: A Scoping Review
Source: J Appl Gerontol. 2025 Nov 14;45(11):2101–16. doi: 10.1177/07334648251394471 (PMC13672951; doi:10.1177/07334648251394471)
Supplement: Supplementary file 1 [file sj-pdf-1-jag-10.1177_07334648251394471.pdf]

Supplementary Table 1. Search strategies for each included electronic databases.

**Ovid Medline(R) ALL<br>1946 – July 12, 2024**	
**Search #**	**Query**
1	exp Decision Support Techniques/
2	(decision* adj3 making* adj3 tool*).ab,kf,ti.
3	"decision aid*".ab,kf,ti.
4	(decision* adj3 guide*).ab,kf,ti.
5	exp Decision Support Systems, Clinical/
6	exp Patient Transfer/ 9907
7	((patient* or care) adj3 (transfer* or transition*)).ab,kf,ti.
8	exp Long-Term Care/
9	"long-term care*".ab,kf,ti.
10	residential facilities/ or assisted living facilities/ or homes for the aged/ or exp nursing homes/
11	(homes adj2 aged).ab,kf,ti.
12	"hospital*".ab,kf,ti.
13	(acute* adj3 care*).ab,kf,ti.
14	1 or 2 or 3 or 4 or 5
15	"emergency department*".ab,kf,ti.
16	"nursing home*".ab,kf,ti.
17	6 or 7 or 8 or 9 or 10 or 11 or 16
18	12 or 13 or 15
19	14 and 17 and 18

**Embase<br>1947 – July 12, 2024**	
**Search #**	**Query**
1	exp decision making/
2	(decision* adj3 making* adj3 tool*).kw,tw.
3	"decision aid*".kw,tw.
4	(decision* adj3 guide*).kw,tw.
5	(patient* adj3 transfer*).kw,tw.
6	(resident* adj3 transfer*).kw,tw.
7	(long-term adj3 care*).kw,tw.
8	(homes adj2 aged).kw,tw.
9	(nursing* adj3 home*).kw,tw.
10	(elder* adj3 home*).kw,tw.
11	(elder* adj3 facilit*).kw,tw.
12	exp assisted living facility/
13	"hospital*".kw,tw.
14	(acute* adj3 care*).kw,tw.
15	1 or 2 or 3 or 4
16	"nursing home*".kw,tw.
17	"emergency department*".kw,tw.
18	5 or 6 or 7 or 8 or 9 or 10 or 11 or 12 or 16
19	13 or 14 or 17
20	15 and 18 and 19
21	limit 20 to (adult <18 to 64 years> or aged <65+ years>)

**CENTRAL<br>Inception - July 12, 2024**	
**Search #**	**Query**
1	exp Decision Making/
2	(decision* adj3 making* adj3 tool*).ti,kw,ab.
3	"decision aid*".ti,kw,ab.
4	(decision* adj3 guide*).ti,kw,ab.
5	(resident* adj3 transfer*).ti,kw,ab.
6	((patient* or care) adj3 (transfer* or transition*)).ti,kw,ab.
7	"long-term care*".ti,kw,ab.
8	exp Assisted Living Facilities/
9	(homes adj2 aged).ti,kw,ab.
10	(nursing* adj3 home*).ti,kw,ab.
11	(elder* adj5 home*).ti,kw,ab.
12	(elder* adj5 facilit*).ti,kw,ab.
13	"hospital*".ti,kw,ab.
14	(acute* adj3 care*).ti,kw,ab.
15	1 or 2 or 3 or 4
16	"nursing home*".ti,kw,ab.
17	"emergency department*".ti,kw,ab.
18	5 or 6 or 7 or 8 or 9 or 10 or 11 or 12 or 16
19	13 or 14 or 17
20	15 and 18 and 19
